# Diagnostic and Clinical Impact of 18F-FDG PET/CT in Staging and Restaging Soft-Tissue Sarcomas of the Extremities and Trunk: Mono-Institutional Retrospective Study of a Sarcoma Referral Center

**DOI:** 10.3390/jcm9082549

**Published:** 2020-08-06

**Authors:** Alessio Annovazzi, Sandra Rea, Carmine Zoccali, Rosa Sciuto, Jacopo Baldi, Vincenzo Anelli, Maria G. Petrongari, Edoardo Pescarmona, Roberto Biagini, Virginia Ferraresi

**Affiliations:** 1 Nuclear Medicine Unit, IRCCS—Regina Elena National Cancer Institute, 00144 Rome, Italy; sandra.rea@ifo.gov.it (S.R.); rosa.sciuto@ifo.gov.it (R.S.); 2 Oncological Orthopaedics Unit, IRCCS—Regina Elena National Cancer Institute, 00144 Rome, Italy; carmine.zoccali@ifo.gov.it (C.Z.); jacopo.baldi@ifo.gov.it (J.B.); roberto.biagini@ifo.gov.it (R.B.); 3 Radiology and Diagnostic Imaging Unit, IRCCS—Regina Elena National Cancer Institute, 00144 Rome, Italy; vincenzo.anelli@ifo.gov.it; 4 Department of Radiation Oncology, IRCCS—Regina Elena National Cancer Institute, 00144 Rome, Italy; mariagrazia.petrongari@ifo.gov.it; 5 Clinical Pathology, IRCCS—Regina Elena National Cancer Institute, 00144 Rome, Italy; edoardo.pescarmona@ifo.gov.it; 6 Medical Oncology 1, IRCCS—Regina Elena National Cancer Institute, 00144 Rome, Italy; virginia.ferraresi@ifo.gov.it

**Keywords:** 18F-FDG PET/CT, soft tissue sarcoma, patient management, staging

## Abstract

Background: Soft-tissue sarcomas (STS) represent a wide heterogeneous class of rare tumors. The exact role ^18^F-fluorodeoxyglucose positron emission/computed tomography (^18^F-FDG PET/CT) in the evaluation of STS is not well established. The aim of the present study was to evaluate how the use of ^18^F-FDG PET/CT in STS could influence patient therapy planning, looking for a possible added value over computed tomography and magnetic resonance imaging—the most used modalities in the study of STS. Differences in SUV_max_ according to histologic subtype and tumor grade were also considered. Methods: a total of 345 consecutive ^18^F-FDG PET/CT scans performed for initial staging (*n* = 171) or for suspected disease relapse (*n* = 174) in 282 patients with STS extracted from the local Information System database were retrospectively reviewed. Results: ^18^F-FDG PET/CT altered therapy planning in 80 cases (16.4% for staging and 29.9% in restaging), both for disease upstaging (58.8%) and downstaging (41.2%) Conclusions: ^18^F-FDG PET/CT could significantly influence management of patients with STS, particularly for restaging.

## 1. Introduction

Soft-tissue sarcomas (STS) are rare tumors including over 80 different histological subtypes with an incidence of 4.7/100,000/year in Europe [1]; the most frequent histotypes, liposarcomas and leiomyosarcomas (LMSs), each have an incidence < 1/100,000/year. The biologic behavior of STS is widely heterogeneous, ranging from locally aggressive lesions to tumors with a high probability of metastatic spread [2]. The mainstay of management is represented by radical surgery [3,4] followed by adjuvant treatment using radiotherapy (RT) or chemotherapy in case of high-grade, deep and >5 cm lesions [5,6]. Preoperative RT and/or chemotherapy represent an option for local control of the tumor [6,7]. Metastatic patients are treated with chemotherapy unless they have exclusive lung involvement for which a surgical approach is usually preferred, particularly in case of a limited number of pulmonary lesions [8]. The choice of the best therapeutic approach is highly influenced by diagnostic imaging. The study of sarcomas is initially performed through physical examination then, if an STS is suspected (e.g., an unexplained deep mass or a superficial lesion of soft tissues with a diameter of >5 cm), a magnetic resonance imaging (MRI) is the first-choice diagnostic tool, ensuring optimal tissue and anatomic characterization of the tumor [9]. Histological characterization of the soft tissue mass is then pursued through multiple core needle or incisional biopsy. If the diagnosis of sarcoma is confirmed, a chest spiral CT scan is indicated to exclude metastatic spread to the lungs. The exact role of ^18^F-FDG PET/CT in the study of STS is still a matter of debate. According to the last European ESMO/EURACAN guidelines for STS [7], the use of ^18^F-fluorodeoxyglucose positron emission/computed tomography (^18^F-FDG PET/CT) can be an option in the pretreatment setting. The NCCN guidelines [10] suggest the use of ^18^F-FDG PET/CT for evaluating the response to chemotherapy [11] and for staging, while they do not propose its regular use for disease relapse unless it is necessary to clarify ambiguous CT or MR findings. Several studies previously demonstrated that ^18^F-FDG PET/CT is a valuable method for staging, predicting prognosis and evaluating the therapy response of STS [12,13].

The specific objective of this retrospective analysis was to evaluate the impact of ^18^F-FDG PET/TC in the modification of the treatment strategy, both in the initial evaluation and in the restaging of STS patients. To this aim, PET/CT findings were compared to MRI for local tumor assessment (performed in almost all patients) and to contrast enhanced computed tomography (ceCT) when available. A second endpoint was to characterize the several histologic subtypes of STS according to the maximum standardized uptake value (SUV_max_), the most widely used semiquantitative PET parameter, which represents a surrogate of lesion glucose avidity, often correlated to biologic aggressiveness of tumors.

## 2. Experimental Section

### 2.1. Patient Population

The present study was approved by the local ethical committee (No. RS1363/20–2367). Due to the retrospective nature of the study an informed consent was not requested. All ^18^F-FDG PET/CT scans performed at our department from 2012 to 2019 for the study of soft-tissue sarcomas were reviewed.

Inclusion eligibility criteria for the analysis were the following:

- Histology-demonstrated soft-tissue sarcomas of the extremities and trunk;
- ^18^F-FDG PET/CT performed at initial staging or for disease restaging (suspect of recurrent disease, doubtful conventional imaging findings and early post-surgical staging);
- Availability of the patient’s clinical information for at least 6 months after PET/CT imaging.

Patients with retroperitoneum and head/neck sarcomas were excluded from the analysis, as well as PET/CT studies performed to monitor the response to treatment.

According to the above criteria, a total of 345 ^18^F-FDG PET/CT scans for initial staging (*n* = 171) or for disease restaging (*n* = 174) in 282 patients were extracted from the local Information System database. Most of the analyzed PET/CT studies (*n* = 330) referred to high-grade sarcomas.

In the staging group, local tumor evaluation was almost always performed by magnetic resonance imaging (MRI). A chest CT or a total body ceCT were available in 21 and 58 patients, respectively.

In the restaging group, PET/CT was performed for:

- Suspected local disease relapse (*n* = 97);
- Early post-surgical staging in patients at high risk for relapse or if a re-excision of the surgical scar was planned (*n* = 25);
- Characterization of suspected lung nodules at CT scan (*n* = 22);
- Suspected metastases in other sites (*n* = 30).

### 2.2. ^18^F-FDG PET/CT Imaging

^18^F-FDG PET/CT scans were performed on a Biograph 16 tomograph (Siemens Medical System, Erlangen, Germany), not equipped with time-of-flight technology. Patients fasted for a minimum of 6 h before the scan and glucose levels below 150 mg/dl were required at time of tracer injection. An average dose of 5 MBq/kg of F-18-FDG was administered intravenously 60 ± 10 min before image acquisition. A non-contrast-enhanced whole-body CT scan was acquired for anatomic localization and attenuation correction (120–140 Kv, 4-mm slice thickness). PET was acquired in standard 3D mode (time-of-flight not supported) immediately after the CT scan, 2–3 min for each bed position. PET images were reconstructed by an ordered subset expectation maximization (OSEM) reconstruction algorithm (TrueX, Siemens Medical System, Erlangen, Germany) with point spread function modeling, using 3 iterations and 21 subsets. After reconstruction, the images were filtered by a Gaussian filter with a full width at half maximum of 4 mm. PET/CT images were reviewed and analyzed both qualitatively (presence/absence of tracer uptake outside sites of physiological accumulation or excretion) and semiquantitatively using the Syngo.via software (Siemens Medical System, Erlangen, Germany) by an experienced nuclear medicine physician. Maximum standardized uptake value (SUV_max_), defined as the pixel with the highest FDG uptake, was measured for the primary tumor and for metastases.

The PET findings were assessed by visual analysis, defining a lesion as “pathologic” when FDG uptake was substantially higher than that of the surrounding background. The specific criteria adopted for some specific conditions are listed below. In the evaluation of local disease relapse, areas of faint focal FDG uptake within the surgical bed were considered as “equivocal”, while a pattern of mild diffuse uptake was judged “unspecific”. Lymph nodes that showed moderately to markedly greater FDG uptake than surrounding tissue were considered positive, regardless of size; on the contrary those showing a faint FDG uptake were indicated as “benign”. Areas of focal bone uptake were considered pathologic, regardless the presence of CT abnormalities on bone window. On the contrary, vague mild bone uptake in the absence of a sclerotic/lytic lesion as well as the presence of uptake associated with degenerative bone alterations were judged as benign findings.

For the analysis of lung metastases, all pulmonary nodules with a size ≥4 mm detected in the CT acquisition of the study were visually examined and judged as “metastatic” at PET scan if they showed an FDG uptake, even slightly higher than that of the surrounding parenchyma. The nodules were also analyzed semiquantitatively by calculating the SUV_max_ target-to-background (T/B) ratio. To this aim, two circle volumes of interest (VOIs) were drawn over each nodule and over the nearby healthy lung parenchyma.

### 2.3. Reference Standard

For validation of PET/CT findings, a combination of imaging follow-up studies (ceCT, PET/CT, MRI) and/or histopathology (if available) was considered as the reference standard. Local disease relapses were nearly all verified by biopsy and/or definitive histology after surgery. Few cases of discordant PET/CT and MRI results were considered “benign” in the absence of significant progression at follow-up imaging. Histological confirmation of metastatic sites was only available in a few cases (except for surgically removed pulmonary metastases), therefore the following criteria were followed for the final diagnosis:

- Lymph nodes: lesions showing a significant FDG uptake regardless of size that increased in number or size on follow-up imaging and/or showed an increase of ^18^F-FDG uptake on follow-up PET/CT were classified as “metastatic”;
- Bone metastases: lytic/sclerotic bone lesions showing definite focal FDG uptake or areas of focal FDG uptake without a corresponding bone alteration that showed increased ^18^F-FDG uptake on follow-up PET/CT and/or that increased in number or size were reported as metastatic; the appearance of lytic/sclerotic changes in follow-up imaging was also considered a sign of malignancy. Non-FDG–avid lytic/sclerotic bone alterations showing no progression in number or size during follow-up were considered as “benign”;
- Lung metastases: pulmonary nodules showing an obvious progression in number and/or size within 6 months were considered as “metastatic”. Lung nodules showing no progression for at least 6 months or that disappeared were considered as “benign”.

In general, all lesions that responded to chemotherapy were also classified as “metastatic”, while those that showed no change or reduction in size and/or of FDG uptake in follow-up imaging without any treatment were classified as “benign”.

### 2.4. Changes in Patient Management

To evaluate the impact of ^18^F-FDG PET/CT scan on patient management and treatment, physicians involved in the study and belonging to the multidisciplinary sarcoma team at our Institution indicated the best therapeutic strategy on the basis of patient clinical data and available diagnostic imaging without knowledge of ^18^F-FDG PET/CT findings. The suggested therapeutic strategy was then compared with the one actually followed by the patient. Each PET/CT study was dichotomized as determining change/no change in patient management.

Change in management was categorized as follows:

1. Non-treatment to treatment;
2. Migration to a different treatment regimen (e.g., from local surgery to systemic chemotherapy);
3. Change in treatment within the same modality (e.g., extension of surgery, neoadjuvant vs. first-line chemotherapy);
4. Treatment to non-treatment.

Confirmation of suspicious CT/MRI findings or evidence of additional disease sites that did not alter clinical management were nevertheless considered in the calculation of the PET/CT diagnostic accuracy. Focal areas of pathologic FDG uptake were checked for a corresponding densitometric finding at non-contrast-enhanced co-registered CT acquisition and with results of ceCT/MRI scans, when available.

### 2.5. Statistical Analysis

All statistical analyses were performed using R software (ver. 3.6.3, Redmond, WA, USA) and Excel datasheets. The Kruskal–Wallis analysis of variance and pairwise comparisons by Mann–Whitney U test were used to test differences in SUV_max_ among sarcoma histological groups. Receiver operating characteristic (ROC) curve analysis was performed to determine the best cutoff of T/B ratio in differentiating malignant from benign pulmonary nodules. Diagnostic sensitivity (Se), Specificity (Sp), accuracy (Acc), positive predictive value (PPV), negative predictive value (NPV) of PET/CT were calculated for lung, bone and lymph node metastases.

## 3. Results

**Figure 1:**
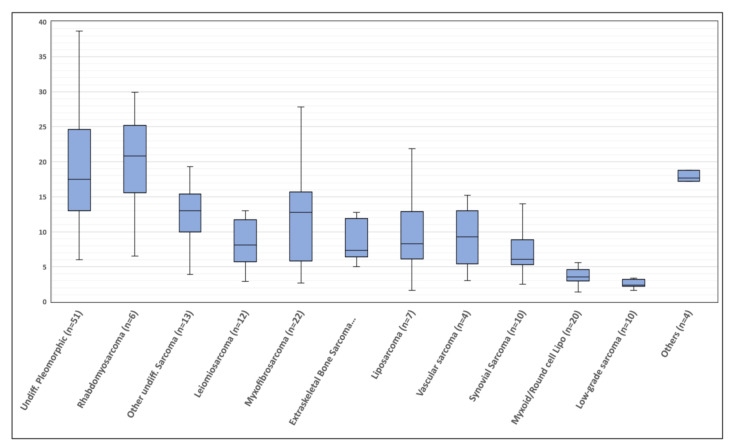
Distribution of standardized uptake value (SUV_max_) values in ^18^F-fluorodeoxyglucose positron emission/computed tomography (^18^F-FDG PET/CT) performed in the initial staging, according to different histological subtypes. Boxplot shows interquartile range (box), median (line within the box) and data range (vertical lines). “*Low-grade sarcoma*” includes: myxoinflammatory fibroblastic sarcoma, low-grade leiomyosarcoma, low-grade liposarcoma and solitary fibrous tumor; “*Others*” includes high-grade solitary fibrous tumor, malignant peripheral nerve sheath tumor and clear cell sarcoma.

All primary tumors were clearly delineated at PET/CT. The SUV_max_ significantly differed among groups (Kruskal–Wallis; *p* < 0.0001) and was globally higher in high-grade vs. low-grade STS (13.8 ± 10.1 vs. 3.4 ± 2.3; *p* < 0.0001), as expected. Pairwise comparison showed that SUV_max_ in all high-grade histological groups was significantly higher compared to synovial sarcoma (7.7 ± 4.3) and myxoid/round cell liposarcoma (3.9 ± 1.9), the latter showing an FDG avidity overlapping that of low-grade sarcomas, despite the high biologic aggressiveness. No statistically significant differences were found among other histological groups, although a trend towards a higher FDG uptake was noticed for undifferentiated pleomorphic sarcoma (20.1 ± 10) and rhabdomyosarcoma (19.8 ± 8.4). The distribution of SUV_max_ according to the histological groups is represented in Figure 1. A great variability in FDG uptake was observed within the same histologic group.

### 3.1. Local Relapse

A total of 88 local disease relapses were confirmed in the 110 patients of the restaging group who performed a PET/CT scan for suspected tumor relapse (*n* = 93) or distant metastases (*n* = 6) or for post-surgical staging (*n* = 11). PET/CT correctly identified 79/88 local disease relapses and 19/25 patients with no evidence of disease. Seven scans were classified as “equivocal”, showing a faint FDG uptake in the surgical bed, four of them corresponded to disease recurrences. ^18^F-FDG PET/CT showed a sensitivity of 95.4% and a specificity of 82.6% if equivocal scans were considered as “positive”, 90.8% and 95.6%, respectively, in case doubtful scans were computed as “negative”.

A head-to-head comparison of PET/CT vs. MRI was available in 90 patients. Results of PET/CT and MRI were accurate and concordant in 69 patients (76.7%). MRI was able to correctly identify a higher number of relapses compared to PET/CT (69 vs. 64), despite a significantly higher rate of false positive results (7 vs. 1). Finally, 4/5 MRI scans judged “equivocal” corresponded to no evidence of disease relapse. All discordant cases were submitted to biopsy to confirm/rule out disease relapse.

### 3.2. Distant Metastases

**Figure 2:**
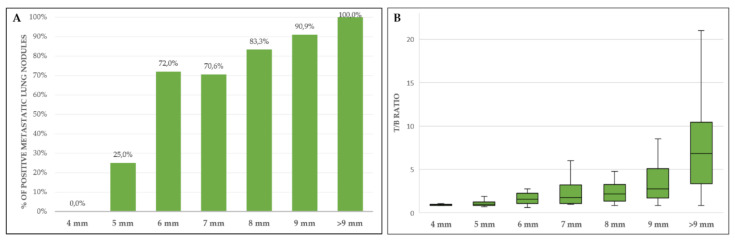
(**A**) Percentage of metastatic lung nodules showing a target-to-background (T/B) ratio > 1.2; (**B**) trend of T/B ratio according to dimensions of metastatic nodules. Boxplot shows interquartile range (box), median (line within the box) and data range (vertical lines).

The presence of one or more sites of metastases was observed in 88 studies (25.5%); the most frequent site was the lung (14.2%), followed by bone (7.5%), lymph nodes (7.2%) and soft tissues (4.6%). Other infrequent sites of metastatic spread were the pleura, liver and abdominal nodules. At least one pulmonary nodule 4 mm was detected on the CT acquisition in 80 patients, for a total of 237 analyzed nodules (193 metastatic and 44 benign). The SUV_max_ target-to-background (T/B) ratio was positively related to the size of the metastatic lung nodules (Pearson r coefficient = 0.657; *p* < 0.0001). On a ROC curve analysis, the optimal T/B ratio to classify a nodule as metastatic was 1.2 (AUC 0.82; CI 0.77–0.88). By using this criterion, PET correctly classified 143/193 metastatic lesions and 43/44 benign nodules with a sensitivity and specificity of 74.1% and 97.7%, respectively. The only false positive result was reported in a patient with sarcoidosis, as confirmed after surgical resection. The rate of positive FDG uptake in metastatic nodules was strongly correlated to their size. PET/CT sensitivity indeed improved significantly when only nodules with a diameter ≥ 6 mm were considered, both on patient-based and on lesion-based analysis (Table 1, Figure 2).

**Figure 3:**
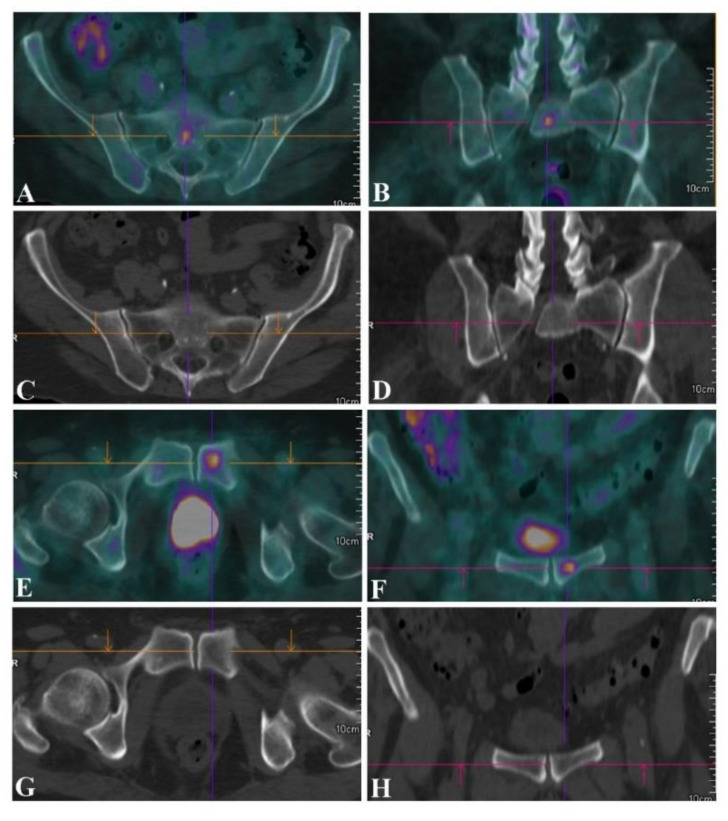
(**A**,**B**; SUV_max_ = 7.1) ^18^F-FDG PET/CT for initial staging in a patient with a rhabdomyosarcoma of the right thigh showing unexpected small FDG-avid bone metastases at the level of the sacrum (**A**,**B**; SUV_max_ = 7.1) and of the left hemipubis (**E**,**F**; SUV_max_ = 7.9), which were not associated with any osteostructural alteration at the (**C**,**D**,**G**,**H**) co-registered CT image. (**A**,**E**) Axial and (**B**,**F**) coronal ^18^F-FDG PET/CT fused images of the sacrum and of the hip, along with the (**C**,**D**,**G**,**H**) corresponding CT images.

The second most frequent site of metastatic spread was the skeleton, as observed in 26 patients for a total of 80 detected metastatic foci. In as many as 41 lesions (51.3%), focal FDG uptake was not related to any osteostructural alteration at co-registered CT scans (Figure 3, Table 1), the remaining ones corresponded to osteolytic (*n* = 34) or osteoblastic (*n* = 5) lesions.

A significantly higher sensitivity of FDG-PET compared to CT scan in the detection of metastatic lymph nodes was observed (96% vs. 56%), with a high rate of false positive findings (*n* = 5; all loco-regional) which, however, was considerably lower than in CT scans (specificity 50% vs. 10%; Table 1).

### 3.3. Impact of ^18^F-FDG PET/CT Scan on Patient Management

^18^F-FDG PET/CT altered patient management in 80 out 345 cases overall (23.2%), 28/171 in the staging group (16.4%) and 52/174 (29.9%) in the re-staging group. A change in the pretreatment TNM stage was observed in 26/170 patients, 19 were upstaged (stage II to IV in 3; IIIa to IV in 5; IIIb to IV in 11) and 7 were downstaged (IV to IIIb in 2; IV to IIIa in 3; IV to Ib in 1; IV to II in 1). In all of these cases, change in patient management was exclusively related to the added value of PET over conventional imaging, both for disease upstaging (58.8%) and downstaging (41.2%). The reasons leading to the change of therapeutic strategy in these patients are summarized in Table 2. In three cases, the change in treatment planning was driven by erroneous PET results: a false positivity for FDG-avid iliac–inguinal lymph nodes and the lack of FDG uptake in a mediastinal lymph node and in an abdominal nodule, both of which were later identified in follow-up studies. In 21 additional cases, the PET/CT scan, as the only whole-body imaging performed, determined a change in patient management for disease upstaging, but in this group PET results were consistent with those of the co-registered CT scans. Details on type of treatment strategy adopted before and after PET/CT results are listed in Table 3 and summarized in Table 4.

## 4. Discussion

The use of ^18^F-FDG PET/CT for staging and restaging STS, with a sensitivity and specificity of up to 98% and 97% in the detection of distant metastases, has been described in several published reports [13,14,15,16]. The assessment of the response to chemotherapy [17,18,19] and prognostication [20] are additional fields of application. Despite the availability of much data from previous reports, the exact framework of the use of PET/CT is yet to be defined in guidelines and in consensus protocols.

The results of the present study ratify the ability of PET/CT to identify a higher number of metastases if compared to CT scans, particularly regarding bone lesions for which only half of FDG-avid lesions had a corresponding osteostructural alteration on CT images. Concerning lung metastases, results confirmed the high positive predictive value of FDG uptake in metastatic pulmonary nodules, but also the low sensitivity in evaluating small metastases [21]. In detail, the results of this series suggest a high reliability of PET/CT in the characterization of nodules greater than eight millimeters, while those of 6–8 mm could cautiously be considered metastatic, particularly if the T/B ratios are close to the cut off value of 1.2. Finally, nodules smaller than six millimeters must always be considered “indeterminate”, unless they have an intense FDG uptake. Similar to other studies [22,23], the accuracy of PET/CT scan in the evaluation of lymph-node involvement resulted less than optimal in this cohort (although significantly higher than CT scans, 80% vs. 42.9%), as it was affected by a high rate of false positive results. Interestingly, all false positive findings (*n* = 5) referred to loco–regional lymph nodes that drained neoplasms characterized by large necrotic areas, resulting in unspecific FDG uptake due to inflammation. Lymph node involvement, although not frequent in STS (range 1.6–12% in literature), is associated with worse prognosis, even in the absence of other metastatic sites [24]. In the present study, this condition occurred in half the cases. A subset of histological subtypes, including synovial sarcoma, rhabdomyosarcoma, clear cell sarcoma, epithelioid sarcoma and angiosarcoma, is more prone to spread to lymph nodes [24], but in our study the greatest number of cases (10/24) occurred in undifferentiated pleomorphic sarcomas (10.4% incidence for this histotype). Concerning local tumor relapse, MRI showed a higher sensitivity vs. PET/CT despite a high rate of false positive results, likely related to unspecific postsurgical or post-actinic tissue alterations. Discordant results between MRI and PET/CT should therefore be managed with close patient follow-upor with biopsy confirmation.

To date, few studies with a limited number of patients have assessed the impact of ^18^F-FDG PET/CT in the management of patients affected by STS. In a study on 89 patients with osteosarcoma and STS Fuglo et al. [22] reported that PET/CT at initial staging determined change in treatment in 18 out of 59 patients (30%), thus avoiding unnecessary surgery and switching to chemotherapy. Similarly, in a study by Elmanzalawy et al. [25], ^18^F-FDG PET/CT at staging demonstrated more lesions when compared to conventional imaging of 12 out of 26 pediatric patients with STS, but altered therapy planning only in five (19%). In a recent report in a wide, mixed population of bone and soft-tissue sarcomas, McPherson et al. [16] demonstrated a potential “added value” of PET/CT over conventional imaging mainly for the detection of additional sites of metastatic disease, although the change in patient management was not addressed in this study. The specific aim of the current retrospective study in a large cohort of patients with STS was to evaluate the potential impact of PET/CT on patient management, which occurred in approximately one-fourth of cases, as supported by the results.

In initial staging, PET/CT exhibited a higher sensitivity in detecting distant metastases compared to conventional imaging, leading to disease upstaging and the consequent switch from a local approach to a systemic chemotherapy. PET/CT was also useful for disease downstaging, due to the lack of FDG uptake in suspicious lesions at CT scan, lung nodules in particular. Surprisingly, an even higher impact (29.9%) of PET/CT was observed for restaging. In this scenario, PET/CT was particularly useful to confirm or rule out local relapse and lung metastases, as well as to detect unsuspected distant metastases (Table 2).

As any retrospective study, limitations are inherent. A selection bias can be assumed for the restaging group for which the choice to perform a PET/CT scan was often linked to equivocal results at conventional imaging—therefore resulting in an upstaging or downstaging of the previous findings. Second, histological confirmation of metastases was only performed in a few cases, as usual in standard clinical practice. Finally, STS are classified as a single entity, but they include over 80 different histological subtypes.

The mainstays of imaging in soft tissue sarcomas are represented by MRI for the assessment of primary tumor and for monitoring disease relapse and CT scan of the thorax for the detection of lung metastases, the most frequent site of metastatic spread in STS. However, the results of the present study suggest that the additional/complementary value of ^18^F-FDG PET/CT over conventional imaging, already demonstrated in previously published reports, does not remain limited to a diagnostic level, but it can be translated into a change in the therapeutic strategy for the patient, in a significant number of cases.

## Figures and Tables

**Table 1:** Accuracy of ^18^F-FDG PET/CT for the detection of distant metastases in the most frequent sites in patients with soft-tissue sarcomas (STS).

Metastatic Site (Total Patients/Lesions Analyzed)	Sensitivity (95% CI)	Specificity (95% CI)	PPV (95% CI)	NPV (95% CI)	Accuracy (95% CI)
Lung (all nodules, ≥4 mm)					
PET/CT (patients *n* = 80)	86.0 (73.3–94.2)	96.7 (82.8–99.9)	97.7 (86.2–99.7)	80.6 (67.5–89.2)	90.0 (81.2–95.6)
PET/CT (nodules *n* = 237)	74.1 (67.3–80.1)	97.7 (87.9–99.9)	99.3 (95.4–99.9)	46.2 (40.3–52.3)	78.5 (72.7–83.5)
Lung (nodules ≥6 mm)					
PET/CT (patients *n* = 70)	89.6 (77.3–96.5)	95.5 (77.2–99.9)	97.7 (86.3–99.7)	80.8 (64.6–90.6)	91.4 (82.3–96.8)
PET/CT (nodules *n* = 181)	88.4 (82.3–93.0)	96.2 (80.4–99.9)	99.3 (95.2–99.9)	58.1 (47.2–68.3)	89.5 (84.1–93.6)
Bone					
PET/CT (patients *n* = 27)	100 (86.8–100)	100 (2.5–100)	100	100	100 (87.2–100)
CT alone	69.2 (48.2–85.7)	–	94.7 (93.3–95.9)	–	66.7 (46.0–83.5)
PET/CT (lesions *n* = 81)	100 (95.5–100)	100 (2.5–100)	100	100	100 (95.6–100)
CT alone	48.8 (37.4–60.2)	–	97.5 (96.9–98.0)	–	48.2 (36.9–59.5)
Lymph nodes					
PET/CT (patients *n* = 35)	96.0 (79.7–99.9)	50.0 (18.7–81.3)	82.8 (72.0–90.0)	83.3 (39.9–97.4)	82.9 (66.4–93.4)
CT alone	56.0 (34.9–75.6)	10.0 (0.3–44.5)	60.9 (50.9–70.0)	8.3 (1.3–38.1)	42.9 (26.3–60.7)

**Table 2:** Reasons for change in patient management due to results of ^18^F-PET/TC over conventional imaging (CT and MRI).

	Staging	Restaging
**FDG-positive occult/suspected disease sites**		
Lung	1	6
Bone	6	2
Lymph nodes	5 *	3
Others (pleura, soft tissues, abdomen)	1	5
Multiple sites	2	5
Local tumor extension/skip metastases	3	2
Local relapse	–	6
**Unconfirmed suspected disease sites (FDG negative)**		
Lung	5	7
Bone	–	1
Lymph nodes	2	5 **
Others (pleura, soft tissues, abdomen)	3	2 ^#^
Local tumor extension/skip metastases	–	0
Local relapse	–	8
Total	28	52

* One false positive due to inflamed iliac-inguinal lymph nodes; ** One false negative mediastinal lymph node; ^#^ One false negative abdominal nodule.

**Table 3:** Impact of ^18^F-FDG PET/CT on the management plan.

Management Plan (Staging)		Management Plan (Restaging)	
**Before PET/CT**	**After PET/CT**	**Before PET/CT**	**After PET/CT**
**Surgery *n* = 56**	Surgery *n* = 44 Surgery + CHT *n* = 1 CHT *n* = 10 PIA+surgery *n* = 1	Surgery *n* = 82	Surgery *n* = 59 Surgery + RT *n* = 1 CHT *n* = 9 RT/CHT *n* = 1 NACT + surgery *n* = 1 FUP/W&S *n* = 11
**Surgery + CHT *n* = 15**	Surgery + CHT *n* = 15	Surgery + CHT *n* = 5	Surgery + CHT *n* = 5
**Surgery + RT *n* = 23**	Surgery + RT *n* = 18 Surgery + CHT *n* = 2 Surgery + RT/CHT *n* = 2 CHT *n* = 1	Surgery + RT *n* = 14	Surgery + RT *n* = 11 CHT *n* = 1 RT *n* = 2
**Surgery + RT/CHT *n* = 9**	Surgery + RT/CHT *n* = 9	Surgery + RT/CHT *n* = 4	Surgery + RT/CHT *n* = 4
**CHT *n* = 11**	Cht *n* = 3 Surgery *n* = 5 Surgery+CHT *n* = 1 Surgery+RT/CHT *n* = 1 NACT + surgery *n* = 1	CHT *n* = 21	CHT *n* = 13 Surgery *n* = 1 Surgery+RT *n* = 1 FUP/W&S *n* = 5 RT *n* = 1
**RT/CHT *n* = 3**	RT/CHT *n* = 3	RT/CHT *n* = 2	RT/CHT *n* = 2
**RT/CHT + Surgery *n* = 6**	RT/CHT + Surgery *n* = 5 CHT *n* = 1		
**NACT+surgery *n* = 45**	NACT+ surgery *n* = 41 CHT *n* = 4	NACT+surgery *n* = 9	NACT+surgery *n* = 7 CHT *n* = 2
**RT *n* = 1**	RT *n* = 1	RT *n* = 6	RT *n* = 5 CHT *n* = 1
**RT + surgery *n* = 2**	RT + surgery *n* = 2	RT + surgery *n* = 1	RT + surgery *n* = 1
		FUP/W&S *n* = 30	FUP/W&S *n* = 7 Surgery *n* = 11 Surgery + CHT *n* = 1 Surgery + RT *n* = 1 CHT *n* = 8 RT/CHT *n* = 1 NACT + Surgery *n* = 1

Legend—CHT—chemotherapy; RT—radiotherapy; NACT—neoadjuvant chemotherapy; FUP—follow-up; W&S—wait-and-see.

**Table 4:** Summary of the contributions of ^18^F-FDG PET/CT in changing patient management.

	Staging (*n* = 34)	Restaging (*n* = 68)
Non-treatment to treatment	–	23
Change in treatment strategy	31	22
Change in treatment within the same modality *	3	7
Treatment to non-treatment	–	16

* extension of tumor resection or additional sites of surgery, chemotherapy regimen (e.g., neoadjuvant to first-line chemotherapy).
